# Honey as a Strategy to Fight *Candida tropicalis* in Mixed-Biofilms with *Pseudomonas aeruginosa*

**DOI:** 10.3390/antibiotics9020043

**Published:** 2020-01-21

**Authors:** Liliana Fernandes, Ana Oliveira, Mariana Henriques, Maria Elisa Rodrigues

**Affiliations:** Centre of Biological Engineering, LIBRO—Laboratório de Investigação em Biofilmes Rosário Oliveira, University of Minho, Campus de Gualtar, 4710-057 Braga, Portugal; lilianafernandes@ceb.uminho.pt (L.F.); anaoliveira@ceb.uminho.pt (A.O.); mcrh@deb.uminho.pt (M.H.)

**Keywords:** *Candida tropicalis*, *Pseudomonas aeruginosa*, biofilms, honey therapy, antifungal agents

## Abstract

Fungal contaminations with *Candida* species are commonly responsible for several infections, especially when associated to bacteria. The therapeutic approach commonly used is being compromised due to microbial resistances of these microorganisms to antimicrobial agents, especially in biofilm. The use of honey as an antimicrobial agent has been emerging as a valuable solution and proving its potential in planktonic and in biofilm cells. This work aims to assess the effect of different honeys on biofilms of *Candida tropicalis* and *Pseudomonas aeruginosa*. The effect of Portuguese heather (PH) and manuka honeys on planktonic growth of *Candida* was initially evaluated by determination of the minimum inhibitory concentrations (MIC). Then, the same effect was evaluated in mixed biofilms, by colony-forming units numeration and fluorescence microscopy. The combinations of honey plus fluconazole and gentamicin were also tested. The results showed that the honeys tested enabled a great reduction of *C. tropicalis*, both in planktonic (12.5% and 25% of MIC for PH and manuka) and in biofilm. In polymicrobial biofilms, the use of PH and manuka honeys was revealed to be a promising choice and an alternative treatment, since they were able to reduce cells from both species. No synergistic effect was observed in antimicrobial combinations assays against polymicrobial biofilms.

## 1. Introduction

Fungal infections are widely recognized as one of the main causes of morbidity and mortality, particularly those infections caused by opportunistic pathogenic fungi, such as the *Candida* species [1]. The incidence of *Candida* infections (Candidosis) has increased remarkably in the last years [1].

On human pathology, *Candida* species have an important role as colonizers of the mucosal membranes of the oral cavity and gastrointestinal tract, as well as normal components of the skin and vaginal flora; so, in normal conditions, *Candida* are non-pathogenic commensal microorganisms in humans [2,3]. However, modifications in human host defenses may lead to a disproportional growth of *Candida* and consequently to a pathogenic colonization by these species [4,5]. In general, transition from commensal to pathogen in *Candida* is facilitated by a series of virulence factors, such as hemolytic activity, secretion of extracellular hydrolytic enzymes (coagulase, phospholipase, and proteases), and producing specific adhesins (for example, fibrinogen and fibronectin), which appear to play an important role in adhesion, penetration, invasion, and destruction of human tissues. Also, the ability of *Candida* to adhere to medical devices or host tissues with the formation of more resistant structures (biofilms) is a particularly important virulence factor [6,7]. In normal environments, biofilm formation represents the most predominant type of microbial growth and is frequently associated to persistent clinical infections [8,9]. While each *Candida* species possesses unique features, biofilms in nature are formed by more than one microbial species, both bacteria and fungi, which confers an even higher resistant phenotype to the biofilm [10]. Although the study of the structure and properties of single-species biofilms is an important step for understanding infectious diseases, the elucidation of communal behavior of microorganisms in biofilms composed of different species may have a high impact for understanding infectious diseases and to develop new therapeutic strategies [10]. Indeed, *Candida* species and *Pseudomonas aeruginosa* microorganisms tend to form polymicrobial biofilms and, as such, they are often responsible for nosocomial infections in immunocompromised individuals [11,12]. 

*Candida albicans* remains as the most prevalent species of these infections, but a clear rise in the proportion of non-*Candida albicans Candida* (NCAC) species has been noted [6,7,13]. According to some epidemiological studies, *Candida tropicalis* stands out in the NCAC species group and is correlated with other forms of *Candida* mortality [14,15,16]. For example, a predominance of NCACs species was observed in the south of America, where *C. albicans* accounted for 40.9% of cases, followed by *C. tropicalis* (20.9%), *C. parapsilosis* (20.5%), and *C. glabrata* (4.9%) [7,17,18]. A study by Kontoyiannis et al. has shown that *C. tropicalis* is more persistent, leading this process to situations of uselessness in cases of infection [14]. This may imply increased virulence and resistance to antifungals compared to *C. albicans*, for example, particularly in the oral cavity [19]. As a consequence of the lack of knowledge on NCAC species virulence, namely in *C. tropicalis*, the rising levels of resistance to the traditional antifungal therapies, and the association of Candidosis to high levels of mortality, there is an urgent need to develop new strategies to fight these infections [1]. In this sense, an alternative approach for the treatment of Candidosis is the use of natural compounds as antifungal agents, among which is honey. Honey is a natural product which has been known for its biological and pharmacological properties for centuries. It has been extensively used in traditional medicine and also complementary medicine because of its antibacterial, antifungal, antimycobacterial, and antiviral activities [20]. These antimicrobial activities may be caused by honey’s acidity (low pH), osmotic effect, high sugar concentration, presence of bacteriostatic and bactericidal factors, increase in cytokine release, as well as immune modulating and anti-inflammatory properties [21,22]. Besides that, one of the most important factors is the presence of hydrogen peroxide (H_2_O_2_), the primary antimicrobial component in most honeys, produced by the enzyme glucose oxidase, and its action consists in the destruction of the essential components of the cells [21,23]. Some types of honey contain additional antimicrobial activity by methylglyoxal (MGO), bee defensin-1, and other bee-derived compounds, such as phenolic compounds of floral origin and lysozyme, among other compounds [23].

The present work aims to evaluate the antimicrobial effect of two different honeys (Portuguese Heather (PH) and manuka) alone, or in combination with a commercial antimicrobial agent, in mixed biofilms of *C. tropicalis* with *P. aeruginosa*. 

## 2. Results 

### 2.1. Susceptibility Testing of Planktonic Populations of C. tropicalis 

The minimum inhibitory concentrations (MIC) and minimum fungicidal concentrations (MFC) of PH and manuka honey against planktonic populations of *C. tropicalis* were determined. A lower MIC was observed for PH (12.5% (w/v)), in comparison to manuka honey (25% (w/v)). Similarly, the MFC recorded for the PH was lower (50% (w/v)) than for manuka honey (>50% (w/v)). 

### 2.2. Effect of Honey in C. tropicalis and P. aeruginosa Biofilms

**Figure 1:**
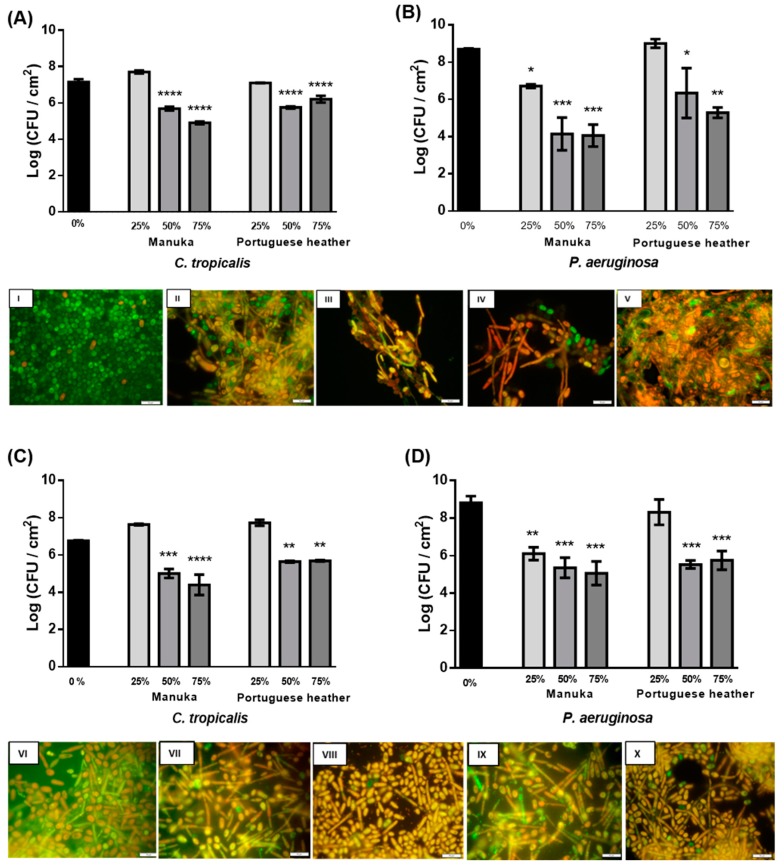
Therapeutic effect of manuka honey and Portuguese heather (PH) on 24 h-old (**A**,**B**) single- and (**C**,**D**) dual-species biofilms formed by (**A**,**C**) *C. tropicalis* and (**B**,**D**) *P. aeruginosa* after 24 h. * *p <* 0.1, ** *p <* 0.01, *** *p <* 0.001, **** *p <* 0.0001 indicates a statistically different reduction in comparison with the respective control. Fluorescence microscopy images of 24 h-old *C. tropicalis* biofilms treated with 0% honey (I), 50% manuka honey (II), 75% manuka honey (III), 50% PH (IV), and 75% PH (V), and images of dual-species (*C. tropicalis* and *P. aeruginosa*) biofilms treated with 0% honey (VI), 50% manuka (VII), 75% manuka (VIII), 50% PH (IX), and 75% PH (X). Live cells were green-stained and dead cells were red-stained. The bar represents 10 μm.

The therapeutic effect of PH and manuka honey (25% (w/v), 50% (w/v), and 75% (w/v)) was assessed on 24 h-old *C. tropicalis* and *P. aeruginosa* single (Figure 1A,B, respectively) and dual-species (Figure 1C,D, respectively) biofilms, by determining biofilm viable cells. The effect of both honeys was monitored at 24 h (Figure 1). The range of honey concentrations tested is in accordance with the results obtained previously for MIC and MFC.

Analyzing the results for 24 h *C. tropicalis* biofilms, a reduction of about 1.5 Log (CFU/cm^2^) relative to the control was observed with both honeys at 50% (w/v). Manuka honey caused the highest cell reduction of about 2 Log (CFU/cm^2^) when used at 75% (w/v) (Figure 1A). For a better understanding of the changes that occurred in these biofilms, after application of honey, they were observed under a fluorescent microscope. By comparing the untreated biofilm image (Figure 1I) with the images after honey administration, an increase in the number of damaged cells (stained in red) is evident. In fact, in the control (Figure 1I), cells were mostly viable (stained in green) and in the yeast form. Comparatively, after the action of both honeys, at 50% (w/v) (Figure 1II,IV) and 75% (w/v) (Figure 1III,V), the cells became clearly damaged, which could be interpreted as a positive action of honey in the infection treatment.

After treatment, it was observed that 75% (w/v) manuka honey had a similar effect of 2 Log (CFU/cm^2^) reduction in *C. tropicalis*, regarding single (Figure 1A) and mixed (Figure 1C) biofilms (*p* < 0.0001). For PH, similar results were obtained with concentrations of 50% (w/v) and 75% (w/v), with a significantly higher reduction in single species biofilms (1 Log (CFU/cm^2^)) (*p* < 0.0001) (Figure 1A) than in mixed biofilms (*p* < 0.01) (Figure 1C).

In relation to *P. aeruginosa*, significant viable cell reductions were also observed after the action of both honeys; nevertheless, the higher reduction (*p* < 0.001) was obtained with manuka honey: 4 and 3 Log (CFU/cm^2^) at 50% (w/v) and 75% (w/v), respectively, in single (Figure 1B) and mixed (Figure 1D) biofilms.

After observation of the mixed biofilms under a fluorescence microscope, it was found that the untreated biofilms had already damaged cells of *C. tropicalis* (Figure 1VI). However, *P. aeruginosa* cells remained viable. It was also found that with 50% (w/v) manuka (Figure 1VII) or PH (Figure 1IX) honeys, the *Candida* cells began to form hyphae. In the treatment with 75% (w/v) manuka (Figure 1VIII) or PH (Figure 1X) honeys, the biofilm was highly damaged in both species. 

### 2.3. Effect of Antimicrobial Combinations Against C. tropicalis and P. aeruginosa Biofilms

**Figure 2:**
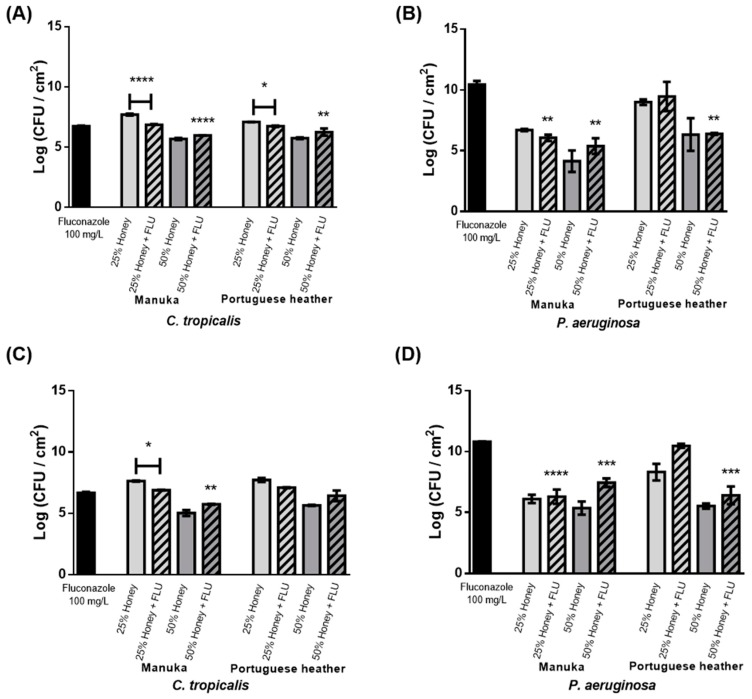
Therapeutic effect of manuka or PH honeys combined with 100 mg L^−1^ of FLU on 24 h-old (**A**,**B**) single- and (**C**,**D**) dual-species biofilms formed by (**A**,**C**) *C. tropicalis* and (**B**,**D**) *P. aeruginosa* at 24 h. * *p <* 0.1, ** *p <* 0.01, *** *p <* 0.001, **** *p <* 0.0001 indicates a statistically different reduction in comparison with the respective control.

The antimicrobial effect of the combination of the different honeys (manuka or PH) at different concentrations (25% (w/v) and 50% (w/v)) with a commercial antifungal agent (100 mg·L^−1^ of fluconazole (FLU)) on single and dual-species 24 h-old biofilms (*C. tropicalis* and *P. aeruginosa*) was monitored at 24 h (Figure 2). Since *P. aeruginosa* has been demonstrated to be susceptible to honey after 6 h of treatment, those concentrations were selected in order to reduce the higher concentrations of honey and the conventional concentrations of FLU. The control assay of 24 h-old single species biofilms is presented in Figure 2A (*C. tropicalis*) and Figure 2B (*P. aeruginosa*).

Regarding the results of Figure 2, it was noted that honey combined with FLU caused higher cell reductions comparatively to treatment with honey at 25% (w/v) (1 Log reduction for manuka honey, *p* < 0.0001; 0.5 Log for PH, *p* < 0.1). Nevertheless, while the combination of 50% (w/v) honey with FLU had no advantage over honey treatment alone, an increased reduction was observed when compared to the effect of FLU alone (*p* < 0.0001 for manuka honey and *p* < 0.01 for heather honey). 

Regarding *C. tropicalis* in mixed biofilms (Figure 2C), and similar to results observed for single biofilms (Figure 2A), less viable cells were recovered after 24 h of combined therapy comparatively to the treatment with manuka honey alone, at 25% (w/v) (*p* < 0.1). Similar results were also observed for *P. aeruginosa*, in single (Figure 2B) and mixed (Figure 2D) biofilms, i.e., when FLU was added, honey substantially increased the number of viable cells in mixed biofilms compared with treatment with honey alone.

**Figure 3:**
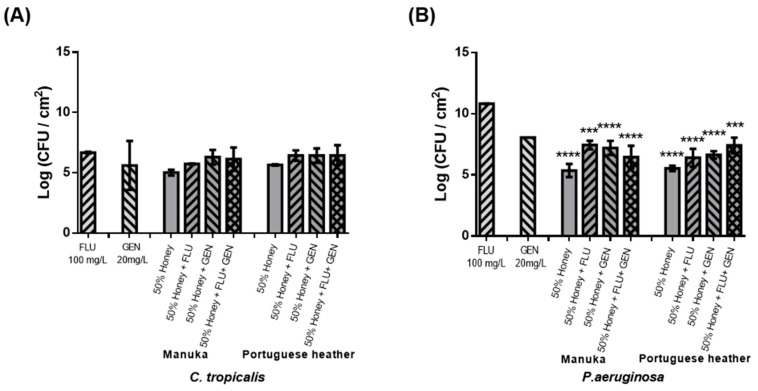
Therapeutic effect of manuka or PH honeys combined with 100 mg L^−1^ of FLU and 20 mg L^−1^ of GEN on 24 h-old dual-species biofilms formed by *C. tropicalis* (**A**) and *P. aeruginosa* (**B**) at 24 h. * *p <* 0.1, ** *p <* 0.01, *** *p <* 0.001, **** *p <* 0.0001 indicates a statistically different reduction in comparison with the FLU.

The efficacy of the honey–antifungal–antibacterial combination was further inspected in preformed dual-species biofilms of *C. tropicalis* (Figure 3A) and *P. aeruginosa* (Figure 3B). The therapeutic effect of the combined action of both honeys at 50% (w/v) with a commercial antifungal (100 mg L^−1^ of FLU) and with a commercial antibiotic (20 mg·L^−1^ of gentamicine (GEN)) was assessed. 

Considering the results obtained with a third antimicrobial agent added (GEN), it is possible to verify more viable cells in biofilms compared to treatment with honey alone, for all conditions tested (Figure 3). 

## 3. Discussion

Analyzing the MIC and MFC values of the two honeys under study for *C. tropicalis*, it was verified that this strain is susceptible to both honeys, especially the PH. Similar results were obtained by Khosravi et al. for *C. tropicalis* (MIC of 38.5% and MFC of 43.7%) [24]. 

The antimicrobial effect of PH and manuka honeys in 24 h biofilms of *C. tropicalis* and *P. aeruginosa* was determined through quantification of viable cells (CFU) per unit of area (cm^2^) (Figure 1). Regarding *C. tropicalis* single biofilms, after 24 h of treatment, none of the honeys at 25% (w/v) caused cell reduction (Figure 1A). This may be a consequence of the fungal cells taking advantage of the lower sugar concentration present in honey at 25% to promote their own growth. Still, significant reductions were obtained with both honeys at 50% (w/v) (about 1.5 Log-reduction (CFU/cm^2^)) and with manuka honey at 75% (w/v) (*p* < 0.0001) (about 2 Log (CFU/cm^2^) (Figure 1A)). A significant reduction was only observed with 75% (w/v) PH (*p* < 0.01). Some properties of honey may be involved in the antimicrobial effect, particularly in the antifungal effect [21,22], such as the presence of metilglioxal (MGO) [25]. an effective antimicrobial agent against planktonic and biofilm cells [26]. The presence of aromatic acids or special proteins with antifungal activity, such as flavonoids, polyphenois, and defensin-1 [25], and the production of H_2_O_2_ by the enzyme glucose oxidase [26]. Indeed, in a previous study, the authors have explored the analysis of these different characteristics of honeys, and the results showed that PH honey was one of the most interesting values indicating the possible high antimicrobial potential (data not shown).

In *C. tropicalis* single and mixed biofilms, after 24 h of treatment, 75% (w/v) manuka honey significantly reduced viable cells in 2 Log (CFU/cm^2^) (*p* < 0.0001) (Figure 1A,C). For PH, similar results were obtained with 50% (w/v) and 75% (w/v) honey, with a significant reduction of 1 Log (CFU/cm^2^) (*p* < 0.0001) (Figure 1A–C). These data suggest that, after 24 h of treatment, manuka honey exerts an antifungal effect both in the presence and absence of *P. aeruginosa*, while PH exerts a slightly higher effect in single species biofilms of *C. tropicalis*. 

The images obtained by fluorescence microscopy (Figure 1I–X), which are only representative of the cell form and structure, revealed a direct relationship between the presence of honey or *P. aeruginosa* and the hyphae. In *Candida* species, the morphological transition involving the formation of hyphae is an important virulence factor, which is associated with cell stress, which could be caused by the presence of *P. aeruginosa* or the several compounds of honey (such as H_2_O_2_ and defensin-1) [27]. Also, from the microscopy images (Figure 1I–X), it was observed that, curiously, the untreated mixed biofilm presented several damaged cells of *C. tropicalis* (Figure 1VI), compared to single biofilms (Figure 1I). This is supported by the experiments carried out by Bandara et al. that investigated the interactions between *Candida species* and *P. aeruginosa*, showing a reduction of 88% (w/v) of *C. tropicalis* after a 24 h-incubation [10]. It was confirmed in the same study that, in general, *Candida* species and *P. aeruginosa* have mutually suppressive effects at all stages of biofilm formation. However, most of the previous studies on interactions between *Candida* species and bacteria in mixed biofilms are concentrated in *C. albicans* and only a few are related to NCAC [10].

Regarding the results obtained for *P. aeruginosa* in single and mixed biofilms, significant reductions could be observed after 6 h of treatment for both honeys at all concentrations tested. In this period of time, the manuka honey allowed for a reduction of the single biofilm to half of the viable cells (*p* < 0.0001) and the PH managed to reduce in average 2–3 Log (CFU/cm^2^). In mixed biofilms, there was also a significant reduction, but it was not as pronounced as in single biofilms (*p* < 0.001). With the results obtained, it can be observed that the honey has a faster and superior antibacterial effect compared to the results obtained for *C. tropicalis*. Comparing the two honeys tested, it was observed that the highest reductions were obtained with manuka honey: 4 and 3 Log (CFU/cm^2^) with 50% (w/v) and 75% (w/v) (*p* < 0.001) in single (Figure 1B) and mixed (Figure 1D) biofilms, respectively. However, the reduction occurred from 25% (w/v) of manuka honey, both in single (*p* < 0.1) (Figure 1B) and in mixed biofilms (*p* < 0.01) (Figure 1D). The effect of lower concentrations of manuka on *P. aeruginosa* was already reported by Cooper and Molan [28]. According to the literature, honey has broad-spectrum antibacterial activity. Regarding the effect of manuka honey on bacteria, a study by Roberts, Moddocks, and Cooper found that honey acts by inhibiting the flogging of bacteria, which limits their mobility and prevents the formation of biofilms [29]. In summary, after 24 h of treatment, honey promoted cell reduction in biofilms of both species simultaneously, suggesting honey as a promising agent for the treatment of polymicrobial infections of *C. tropicalis* and *P. aeruginosa*. Very few treatments were reported as being able to reduce more than one species in a mixed biofilm.

The effect of the combination of honey (PH or manuka honeys at 25% (w/v) and 50% (w/v)) with FLU (100 mg L^−1^) was then evaluated in single and mixed biofilms (Figure 2). For *C. tropicalis*, the cell reduction obtained with the combination (honey and FLU) was higher than with only honey at 25% (w/v) (1 Log reduction for manuka honey, *p* < 0.0001; 0.5 Log for PH, *p* < 0.1) (Figure 2A). Conversely, combinations of honey at 50% (w/v) and FLU had no advantage over honey treatment alone; nevertheless, an improved reduction was observed when compared to the effect of FLU alone (*p* < 0.0001 for manuka honey and *p* < 0.01 for heather honey). Comparing results obtained for honey combined with an antifungal agent, it was observed that it had advantages over single honey or antifungal treatment. The combined treatments allowed for a 50% reduction of the dosage of antifungal typically required in clinical settings, i.e., 200 mg L^−1^. 

In mixed biofilms, a slight reduction in biofilm was observed after 12 h compared to the individual treatment with either honey or FLU. After 24 h of combinational therapy, a significant reduction was only obtained in comparison to treatment with manuka honey alone at 25% (w/v) (*p* < 0.1) (Figure 2C), similar to what was observed for single biofilms (Figure 2A). This demonstrates that the combination of *C. tropicalis* and *P. aeruginosa* is indifferent to treatment with honey and FLU, and that *C. tropicalis* remains less tolerant to FLU. Furthermore, adding FLU to honey substantially increased the number of *P. aeruginosa* viable cells in mixed biofilms (Figure 2D) compared with treatment with only honey. 

In summary, honey by itself allows for a superior reduction in comparison with the combination of honey and FLU in all conditions tested in mixed and single biofilms, with the exception of 25% (w/v) honey. *C. tropicalis* remains highly resistant to FLU, even when combined with honey, both in single and mixed biofilms. This is in accordance with several studies stating that resistance to FLU is increasing in clinical isolates of this species [29]. 

Considering the results previously obtained, a third antimicrobial agent was added (GEN) to the combinations tested. So, combinational therapy was evaluated using honey 50% (w/v), FLU 100 mg L^−1^, and GEN 20 mg L^−1^. However, this resulted in an increased number of viable cells in biofilms compared to treatment with honey alone, for all conditions tested (Figure 3). Indeed, the combination of these three elements had no advantage over treatment with honey alone, both for the fungal and bacterial species. The interaction between FLU and GEN was investigated by Thomas et al. as a treatment of pre-formed biofilms of 4, 8, and 12 h of *C. albicans*, where synergism was observed only against pre-formed biofilms of 4 and 8 h, with no synergism observed at 24 h [30].

## 4. Material and Methods

### 4.1. Microorganisms and Culture Conditions

*C. tropicalis* ATCC 750 and *P. aeruginosa* DSM 22644 were stored at –80 ± 2 °C in broth medium with 20% (v/v) glycerol. Prior to each assay, *C. tropicalis* and *P. aeruginosa* strains were subcultured from the frozen stock preparations onto Sabouraud Dextrose Agar (SDA) and Tryptic Soy Agar (TSA) plates, respectively. SDA and TSA were prepared from Sabouraud Dextrose Broth (SDB; Liofilchem, Roseto degli Abruzzi, Italy) or Tryptic Soy Broth (TSB; Liofilchem, Roseto degli Abruzz, Italy), supplemented with 2% (w/v) agar (Liofilchem, Roseto degli Abruzzi, Italy). The plates were then incubated aerobically at 37 °C for 18–24 h. 

Pure liquid cultures (pre-inocula) of *C. tropicalis* were maintained in SDB, whereas *P. aeruginosa* was grown overnight in TSB. For planktonic and biofilm assays, 0.22 µm filter-sterilized Roswell Park Memorial Institute (RPMI) 1640 medium (Gibco^®^ by Life Technologies ^TM^, Grand Island, NY, USA) at pH 7.0 was used. Unless otherwise stated, all rinse steps were performed either by using 0.9% (w/v) saline solution (NaCl; J.T. Baker, Deventer, The Netherlands) or ultrapure (UP) sterile water.

### 4.2. Antimicrobial Agents

Stock solutions of two commercial antimicrobial agents, Fluconazole (FLU, Sigma-Aldrich, St. Louis, MO, USA) and Gentamicin sulfate (GEN, Sigma-Aldrich, St. Louis, MO, USA) were prepared and stored according to the manufacturer’s instructions. Also, two different honeys, Portuguese heather honey (raw dark amber honey whose main plant nectar is heather (30%, APISMaia company (Porto Portugal)) collected by a beekeeper in the North of Portugal) and manuka (commercial Medihoney^®^) were stored at 4 °C, and the dilutions were prepared with RPMI 6420 medium. All the concentrations of different antimicrobial agents (natural and commercial) tested in this work are presented in Table 1. 

### 4.3. Planktonic Antimicrobial Susceptibilities

Susceptibilities of *C. tropicalis* planktonic-cell cultures were evaluated by determining the minimum inhibitory concentration (MIC) and the minimum fungicidal concentration (MFC). The MIC values were determined according to standard European Committee on Antimicrobial Susceptibility Testing (EUCAST), through the broth microdilution method [32]. Briefly, the initial cell concentration for both microorganisms was adjusted for 1 × 10^6^ CFU/mL and dispensed into 96-well plates in a proportion of 1:2 (the final inoculum concentration was 5 × 10^5^ CFU/mL) with the working antimicrobial solutions (previously diluted in RPMI 1640 broth with double of the desired final concentration). Wells containing only broth medium (antimicrobial-free medium) were used as negative controls and wells containing *C. tropicalis* culture without antimicrobial agent were used as positive controls. Plates were incubated overnight at 37 °C. MIC was obtained by visual observation of the turbidity gradient. The minimum concentration where growth inhibition occurs is equivalent to the MIC value.

For the determination of MFC values, 10 μL was removed from each well of the microdilution trays, after incubation, and plated onto SDA plates and incubated at 37 °C. The lowest antimicrobial concentration that yielded no colony growth after 12–24 h was considered as the MFC.

### 4.4. Biofilms Antimicrobial Susceptibilities

Biofilms were developed according to the modified microtiter plate test proposed by Stepanović et al., with some modifications [33]. Briefly, different cultures were centrifuged twice (3000 *g*, 4 °C, 10 min) and the pellet was resuspended in RPMI 1640, until reaching 1 × 10^7^ cells/mL. Yeast cells (*C. tropicalis*) were enumerated by microscopy using a Neubauer counting chamber. Bacteria (*P. aeruginosa*) concentration was adjusted to 0.13 (corresponding between 2–3 × 10^−8^ CFU·mL^−1^), using an ELISA microtiter plate reader with a wavelength of 640 nm (Sunrise-Basic Tecan, Männedorf, Switzerland). For mixed-species cultures, a combination of 50% of the suspended inoculum of each species was used. 

The cellular suspensions were transferred, under aseptic conditions, to 96-well flat tissue culture plates (polystyrene, Orange Scientific, Braine-L’Alleud, Belgium) (200 μL per well). To promote biofilm formation, microtiter plates were incubated aerobically for 24 h on a horizontal shaker at 120 rpm and 37 °C. 

The effect of each honey (PH or manuka) alone was evaluated in single and mixed-species biofilms of *C. tropicalis* and *P. aeruginosa*. For this, 24 h-old biofilms were exposed to increasing concentrations of each agent (25% (w/v), 50% (w/v), and 75% (w/v)). Briefly, after biofilm formation, 200 μl of cell suspension were replaced by the antimicrobial solutions prepared at 2-fold the desired concentration. Plates were then incubated aerobically at 37 °C for 24 h. After 6, 12, and 24 h, the treated biofilms were removed to assess biofilm-cells cultivability through CFU enumeration. 

After biofilm formation, the wells were washed twice with saline solution after discarding the planktonic fraction. In order to estimate the number of cultivable biofilm-entrapped cells in single- and mixed-species, the microdrop technique was used. Briefly, 200 μL of fresh saline solution was added to each well and the biofilms were scraped. The resulting biofilm-cells suspensions were then serially diluted in saline solution and plated onto non-selective agar (SDA for *C. tropicalis* and TSA for *P. aeruginosa* pure cultures) plates. Selective agar was also used for colony-forming units (CFU) determination of *Candida* species (SDA supplemented with 20 mg L^−1^ gentamycin (GEN), to suppress the growth of *P. aeruginosa*) and *P. aeruginosa* (*Pseudomonas* Isolation Agar, (PIA)). Agar plates were incubated aerobically at 37 °C for 24 h for cultivable cell counting. Values of cultivable sessile cells were expressed as Log CFU per area (cm^2^). All negative (wells containing only broth medium) and positive (wells containing *C. tropicalis*, *P. aeruginosa*, *C. tropicalis*, and *P. aeruginosa* cultures without antimicrobial agents) controls were performed.

### 4.5. Combinatorial Effect of Antimicrobial Agents (Honey and Commercial Agents) on Biofilms

The combinatorial effect of honeys (25% (w/v) and 50% (w/v) of PH or manuka honey with 100 mg L^−1^ FLU was assessed against 24 h-old *C. tropicalis* biofilms, following a procedure similar to the individual application of the antimicrobials, and against 24 h-old dual-species (*C. tropicalis* and *P. aeruginosa*) biofilms. Also, the triple combinatorial effect of antimicrobial agent (100 mg L^−1^ FLU and 20 mg L^−1^ GEN) and 50% (w/v) of honey (PH or manuka) was assessed against 24 h-old dual-species (*C. tropicalis* and *P. aeruginosa*) biofilms. Biofilm cells were removed after 6, 12, and 24 h to assess biofilm-cells cultivability through CFU enumeration. Also, all negative (wells containing only broth medium) and positive (wells containing *C. tropicalis*, *P. aeruginosa*, *C. tropicalis*, and *P. aeruginosa* cultures without antimicrobial agents) controls were performed.

### 4.6. Cell Viability Assessment of Biofilm-Embedded Cells 

In order to evaluate the cell morphology and viability of polymicrobial biofilms after treatment in a qualitative way, the Live/Dead^®^ BacLight™ Bacterial Viability Kit (Molecular Probes, Leiden, The Netherlands) was employed. Basically, biofilms were formed on polystyrene coupons, as described above, and were then stained for 15 min in the dark with a mixture of the SYTO 9 and Propidium Iodide, both prepared at 3 μl/mL in saline solution. For microscopic observation, an Olympus BX51 microscope fitted with fluorescence illumination was used. The optical filter combination consisted of 470 to 490 nm in combination with 530 to 550 nm excitation filters. 

### 4.7. Statistical Analysis 

Data were analyzed using the Prism software package (GraphPad Software version 6.01 for Macintosh). One-way analysis of variance (ANOVA) tests were performed, and means were compared by applying Tukey’s multiple comparison test. The statistical analyses performed were considered significant when *p* < 0.1. For all assays, at least three independent experiments were carried out in triplicate.

## 5. Conclusions

The increasing incidence of *Candida*-associated infections requires the discovery of more efficient new antifungal therapies, with less adverse effects. In this scope, honey emerges as a potential antifungal agent. Here, the antifungal effect of different concentrations of PH and manuka honeys was evaluated in *C. tropicalis*. A reduction of *C. tropicalis* cell growth in both planktonic and biofilm state was observed with honey treatment. Comparing both honeys tested, for biofilm culture, manuka had a higher effect than PH. 

However, understanding the behavior of *Candida* species in polymicrobial biofilms is an important step in the clinical context and for the selection of the most efficient treatment. Because of this, the effect of both honeys was assessed on mixed biofilms of *C. tropicalis* and *P. aeruginosa*. The honeys were able to reduce both species in the mixed biofilm and were demonstrated to be a promising alternative for the treatment of infections caused by mixed species biofilms. The combinations, honey–antifungal and honey–antifungal–antibiotic, were also tested but without positive results. Other antifungal and antimicrobial agents need to be tested to understand the feasibility of using these combinations in therapy. 

Overall, the results obtained here highlight the potential of honey as an alternative therapy for controlling infections induced by *C. tropicalis*, especially when associated to bacteria. The use of a natural product such as PH honey may be used in clinical practice, especially in skin applications, to prevent or even treat *C. tropicalis* and *P. aeruginosa* infections. 

## Figures and Tables

**Table 1:** Tested concentrations of honey (Portuguese heather (PH) and manuka), commercial antifungal agent (FLU) and commercial antibiotic agent (GEN)**.**

Antimicrobial Agents		Concentration
**Honeys**	**Manuka** **PH**	6% (w/v) 12.5% (w/v) 25% (w/v) 50% (w/v) 75% (w/v)
**FLU**		100 mg L^−1^ *
**GEN**		20 mg L^−1^ *

* Values of concentrations selected in a previous study [31].
